# The Association between Serum Uric Acid and Bone Mineral Density in Older Adults

**DOI:** 10.1155/2020/3082318

**Published:** 2020-06-29

**Authors:** Xiaocong Yao, Lin Chen, Huihui Xu, Zhongxin Zhu

**Affiliations:** ^1^Department of Osteoporosis Care and Control, The First People's Hospital of Xiaoshan District, Hangzhou, Zhejiang 311200, China; ^2^Department of Immune and Rheumatology, The First People's Hospital of Xiaoshan District, Hangzhou, Zhejiang 311200, China; ^3^Zhejiang Chinese Medical University, Hangzhou, Zhejiang 310053, China; ^4^Institute of Orthopaedics and Traumatology of Zhejiang Province, Hangzhou, Zhejiang 310053, China

## Abstract

**Objectives:**

Uric acid has been found to be potentially protective in bone metabolism. We investigated the relationship between serum uric acid (sUA) and lumbar bone mineral density (BMD) among 4156 participants aged 60 years and over from the National Health and Nutrition Examination Survey (NHANES).

**Methods:**

To estimate the association between sUA and lumbar BMD, multivariate logistic regression analyses were conducted. Fitted smoothing curves and generalized additive models were also performed.

**Results:**

We found sUA positively correlated with lumbar BMD after adjusting for other confounders. On subgroup analyses, stratified by sex and race/ethnicity, the positive correlation of sUA with lumbar BMD remained in both men and women, as well as in whites and Mexican Americans, but not in blacks. In blacks, the association of sUA with lumbar BMD was an inverted *U*-shaped curve (inflection point: 7.5 mg/dL).

**Conclusions:**

Our study revealed a positive relationship between sUA and lumbar BMD among most old adults. This association followed an inverted *U*-shaped curve among blacks.

## 1. Introduction

Over the last decade, the proportion of elderly individuals in the general population has been steadily increasing worldwide. Currently, individuals over the age of 60 years make up >11% of the global population, with this proportion expected to increase to about 22% by 2050 [1]. As the population ages and with lifestyle changes, the number of older individuals with osteoporosis is set to increase dramatically in the coming decades. The International Osteoporosis Foundation estimates that one in three women and one in five men over the age of 50 years will experience an osteoporotic fracture [2]. Owing to the healthcare costs, morbidity, and mortality associated with fragility fractures, the clinical and public health systems will be under tremendous pressure. Therefore, understanding the risk factors for lower bone mineral density (BMD) is essential for the prevention, early diagnosis, and management of osteoporosis.

The clinical assessment of risk factors of osteoporosis contributes to identifying candidates who would benefit from BMD screening, using dual-energy X-ray absorptiometry (DEXA), for effective early intervention to reduce the incidence of fragility fractures. In this regard, there is ongoing research interest in identifying novel, as well as less well-studied biomarkers of osteoporosis, such as serum uric acid (sUA).

Hyperuricemia is a major pathogenic factor for gout, with UA having been considered as a metabolic waste product for a long time [3]. However, there is growing evidence that UA, as an antioxidant, might play protective roles in cancer [4] and nervous system diseases, such as dementia [5], Parkinson's disease [6], Alzheimer's disease [7], multiple sclerosis [8], and amyotrophic lateral sclerosis [9]. Moreover, due to its antioxidant properties, UA is also considered to contribute to greater BMD by inhibiting osteoclastic bone resorption and promoting osteoblastic differentiation [10].

Recently, studies are being focused on the relationship between sUA and BMD, but with controversial findings having been reported in this limited body of evidence. Specifically, while a higher sUA level was associated with greater BMD [10], other studies did not identify a protective effect of higher sUA on BMD [11–13]. Accordingly, our aim in this study was to evaluate the relationship between sUA and BMD using a representative sample of older adults from the National Health and Nutrition Examination Survey (NHANES).

## 2. Materials and Methods

### 2.1. Statement of Ethics

The study was approved by the ethics review board of the National Center for Health Statistics, and written consent was obtained from each participant.

### 2.2. Study Population

The NHANES is a representative survey of the national population of the United States (US), providing multitudinous information about the nutrition and health of the general US population using a complex, multistage, probability sampling design [14].

Our analysis was based on data from 1999–2006, which represent three cycles of the NHANES. After exclusion of participants with missing sUA data (*n* = 1154), lumbar BMD data (*n* = 930), participants with cancer (*n* = 862), and participants who received allopurinol during a 1-month period prior to the survey date (*n* = 75), a total of 4156 participants ≥60 years of age were included in our analysis.

### 2.3. Variables

The exposure variable of this study was sUA. Between 1999 and 2001, the 704 Multichannel Analyzer or Roche Hitachi Model 917 were used to measure sUA, with the Beckman Synchron LX20 used since 2002. The outcome variable was lumbar BMD, measured by DEXA. The following categorical variables were included in our analysis as covariates: sex, race/ethnicity, level of education, alcohol consumption, smoking behavior, physical activity, and use of calcium supplementation. The continuous covariates were included in our analysis: age, poverty to income ratio, waist circumference (WC), C-reactive protein (CRP), blood urea nitrogen (BUN), total protein, total cholesterol, serum phosphorus, and serum calcium. The detailed information on sUA, lumbar BMD, and covariates are publicly available at http://www.cdc.gov/nchs/nhanes/.

### 2.4. Statistical Analysis

We performed a weighted and variance estimation analysis to account for the marked variance in our data set. A weighted multivariate logistic regression model was used to evaluate the association between sUA and lumbar BMD. We used the weighted *χ*2 test for categorical variables or the weighted linear regression model for continuous variables to calculate the difference among each group. The subgroup analysis was performed by stratified multivariate regression analysis. Furthermore, smooth curve fittings and generalized additive models were used to address the nonlinear relationship between sUA and lumbar BMD. For nonlinear models, the inflection point in the relationship between sUA and BMD was calculated using a recursive algorithm, with a two-piecewise linear regression model conducted on both sides of the inflection point, when nonlinearity was detected. All analyses were performed with package *R* (http://www.R-project.org) and EmpowerStats (http://www.empowerstats.com), with a *P* value <0.05 considered statistically significant.

## 3. Results

A total of 4156 participants, 60–85 years of age, were included in our analysis, with the weighted characteristics of the participants subclassified based on sUA quartiles (Q1:1.5–4.5 mg/dL; Q2: 4.6–5.4 mg/dL; Q3: 5.5–6.4 mg/dL; and Q4: 6.4–13.7 mg/dL), as shown in Table 1. There were significant differences in baseline characteristics between the sUA quartiles, with the exception of the level of education, and the income to poverty ratio. Compared to the other subgroups, participants in the highest sUA quartile were more likely to be men; blacks, with lower values of total cholesterol and serum phosphorus and higher WC, BUN, CRP, total protein, and serum calcium levels, and lumbar BMD.

The results of the multivariate regression analyses are presented in Table 2. In the unadjusted model, sUA was positively correlated to lumbar BMD (*β* = 0.030, 95%CI: 0.026–0.034, *P* < 0.001). After adjustment for confounders, this positive association was still present in model 2 (*β* = 0.019, 95%CI: 0.015–0.022, *P* < 0.001) and model 3 (*β* = 0.010, 95%CI: 0.006–0.014, *P* < 0.001). After converting sUA from a continuous variable to a categorical variable (quartiles), individuals in the highest quartile had a 0.023 g/cm^2^ greater BMD than those in the lowest sUA quartile.

**Figure 1:**
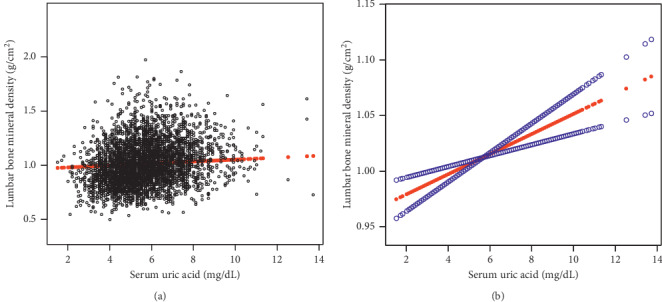
The association between serum uric acid and lumbar bone mineral density. (a) Each black point represents a sample. (b) Solid rad line represents the smooth curve fit between variables. Blue bands represent the 95% of confidence interval from the fit. Age, sex, race/ethnicity, waist circumference, education, income poverty ratio, physical activity, smoking behavior, alcohol consumption, blood urea nitrogen, C-reactive protein, total protein, total cholesterol, serum phosphorus, serum calcium, and calcium supplementation use were adjusted.

**Figure 2:**
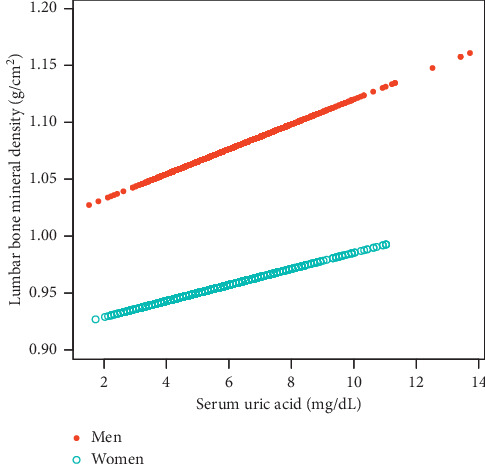
The association between serum uric acid and lumbar bone mineral density stratified by sex. Age, race/ethnicity, body mass index, education, income poverty ratio, physical activity, smoking behavior, alcohol consumption, blood urea nitrogen, C-reactive protein, total protein, total cholesterol, serum phosphorus, serum calcium, and calcium supplementation use were adjusted.

**Figure 3:**
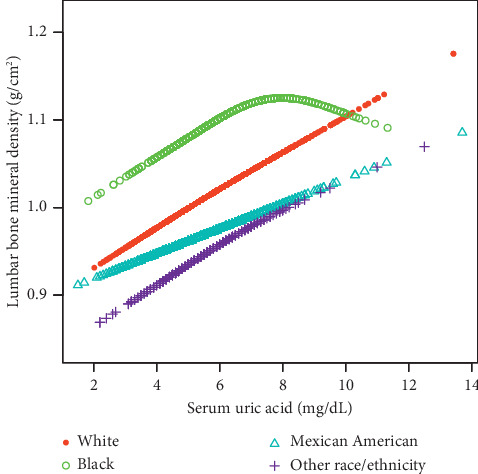
The association between uric acid and lumbar bone mineral density stratified by race/ethnicity. Age, sex, body mass index, education, income poverty ratio, physical activity, smoking behavior, alcohol consumption, blood urea nitrogen, C-reactive protein, total protein, total cholesterol, serum phosphorus, serum calcium, and calcium supplementation use were adjusted.

On subgroup analyses, stratified by sex and race/ethnicity, reported in Table 2, the positive correlation of sUA with lumbar BMD remained in both men (*β* = 0.014, 95%CI: 0.008–0.020, *P* < 0.001) and women (*β* = 0.008, 95%CI: 0.003–0.014, *P*=0.001), as well as in whites (*β* = 0.012, 95%CI: 0.006–0.017, *P* < 0.001) and Mexican Americans (*β* = 0.010, 95%CI: 0.001–0.018, *P*=0.023), but not in blacks. Smooth curve fittings and generalized additive models used to characterize the nonlinear relationship between sUA and lumbar BMD are shown in Figures 1–3. Among blacks, the association between sUA and lumbar BMD was an inverted *U*-shaped curve, with the point of inflection identified using a two-piecewise linear regression model, at 7.5 mg/dL (Table 3). For a sUA <7.5 mg/dL, every 1 mg/dL increase in sUA was associated with a 0.018 g/cm^2^ greater lumbar BMD (95%CI: 0.004–0.031); by comparison, for individuals with a sUA >7.5 mg/dL, a 1 mg/dL increase in sUA was associated with a 0.026 g/cm^2^ decrease in lumbar BMD (95%CI: −0.055–0.004).

## 4. Discussion

Our multivariate logistic regression analyses indicated an elevated sUA correlated with a greater lumbar BMD. However, on subgroup analysis, we identified a nonlinear relationship between sUA and lumbar spine BMD among blacks, with a point of inflection at 7.5 mg/dL.

Over the past few decades, the prevalence of high levels of hyperuricemia has been increasing [15, 16]. Hyperuricemia is a key causal factor of gout, as well as associated with a wide range of conditions, such as chronic kidney disease, obesity, and hypertension [17, 18]. On the other hand, higher sUA might play a beneficial role in some conditions, including osteoporosis [10]. Among our representative US population, a higher sUA was associated with a greater lumbar BMD in most old adults. Considering this association, sUA could provide a potential predictive biomarker for osteoporosis. Thus, measurement of the sUA level could provide a screening tool for osteoporosis to guide therapeutic interventions, as well as to avoid an overcorrection of sUA among patients with osteoporosis.

Currently, clinical studies regarding the relationship between sUA and BMD among older adults are limited and controversial. Four cross-sectional studies from China reported a positive correlation between a higher sUA and greater BMD among postmenopausal women and older adults [19–22]. This conclusion was supported by other studies from Asia [23–25], as well as from the Netherlands and Italy [26, 27]. However, other studies did not support this conclusion. Specifically, a cross-sectional study from China reported a positive correlation between the sUA and BMD among postmenopausal women (*n* = 4256), but not men (*n* = 943) [28]. Furthermore, a Mendelian randomized study including 226 Chinese older men and 1108 postmenopausal women did not find a causal association between sUA and BMD, measured at various sites [29]. A cohort study conducted in the US did not identify a correlation between a high sUA and the incidence of hip fractures among women, while the association between the sUA level and hip fractures in men followed a *U*-shaped curve [30]. Heterogeneity between these studies, including differences in the study design, study sample, distribution of race, and the confounding variables controlled for, may explain the controversial findings between studies. In our study, we controlled for WC rather than body mass index (BMI), as WC is a stronger predictor of sUA and BMD than BMI, as recently reported [31]. We note that we further performed subgroup analyses for more appropriate representation of the data set as recommended by the STROBE statement [32]. Our findings indicated that a higher sUA was associated to a higher lumbar BMD in both older men and women, with this association being more pronounced among men. Moreover, our subgroup analyses stratified by race/ethnicity revealed, for the first time, an inverted *U*-shaped association between sUA and lumbar BMD among blacks. A higher prevalence of hyperuricemia and gout has also been reported among blacks than whites and Mexican Americans [15, 33]. Differences in genetic risk factors, obesity status, alcohol consumption, and other factors may provide a possible explanation for noted race-specific differences. Further prospective studies with large study samples are required to clarify the association between sUA and BMD among elderly individuals of the black race.

As we used a nationally representative sample, the results of our study are highly relevant to the whole population. Moreover, our large sample size allowed us to perform subgroup analyses and is the first study, to our knowledge, to have reported on the relationship between sUA and lumbar BMD among blacks. However, it is important to acknowledge the limitations of our study. Foremost, it is the cross-sectional design of our study, which limits the inference of a causal correlation between sUA and lumbar BMD among older adults. Therefore, further basic mechanistic research and large sample prospective studies are warranted to identify the exact mechanism of the association between sUA and BMD. Second, we excluded individuals with malignancy from our study sample as malignancy may have a significant influence on lumbar BMD. Third, there remains the possibility of bias caused by other potential confounding factors that we did not adjust for.

## 5. Conclusions

Our study revealed a positive relationship between sUA and lumbar BMD among most old adults. This association followed an inverted *U*-shaped curve (inflection point: 7.5 mg/dL) among blacks. Measurement of sUA may provide a responsive biomarker for the early identification of osteoporosis and to guide treatment.

## Figures and Tables

**Table 1:** Weighted characteristics of the study population based on serum uric acid quartiles.

Serum uric acid (mg/dL)	Total	Q1 (1.5–4.5)	Q2 (4.6–5.4)	Q3 (5.5–6.4)	Q4 (6.4–13.7)	*P* value
Age (years)	69.21 ± 7.28	68.97 ± 7.19	69.20 ± 7.25	68.84 ± 6.97	69.80 ± 7.69	0.0125
Sex (%)						<0.0001
Men	42.96	20.22	37.42	51.80	60.32	
Women	57.04	79.78	62.58	48.20	39.68	
Race/ethnicity (%)						<0.0001
Non-Hispanic white	79.30	79.57	80.06	79.36	78.26	
Non-Hispanic black	8.60	5.75	7.87	8.90	11.61	
Mexican American	3.91	4.97	4.54	3.39	2.83	
Other race/ethnicity	8.20	9.70	7.53	8.35	7.30	
Waist circumference (cm)	100.44 ± 14.17	92.87 ± 13.04	99.10 ± 13.51	103.08 ± 13.14	106.06 ± 13.54	<0.0001
Level of education (%)						0.7976
Less than high school	29.34	29.97	28.54	30.04	28.84	
High school	29.42	30.45	28.81	28.23	30.22	
More than high school	41.24	39.58	42.65	41.73	40.94	
Income to poverty ratio	2.79 ± 1.45	2.72 ± 1.47	2.82 ± 1.45	2.81 ± 1.44	2.79 ± 1.44	0.4547
Physical activity (%)						0.0081
Sedentary	25.13	27.89	21.62	24.06	27.03	
Low	25.01	27.07	23.49	24.39	25.20	
Moderate	16.41	14.45	17.93	17.29	15.90	
High	26.30	24.99	28.84	27.34	24.03	
Not recorded	7.14	5.61	8.10	6.93	7.84	
Smoking behavior (%)						<0.0001
None	47.00	53.18	46.80	47.30	41.20	
Past	39.07	31.81	38.36	37.73	47.76	
Current	13.93	15.01	14.83	14.97	11.04	
Alcohol consumption (%)						<0.0001
Nondrinker	47.61	50.37	46.51	48.83	44.83	
Moderate alcohol use	28.99	33.83	30.44	26.31	25.83	
High alcohol use	23.40	15.80	23.05	24.86	29.34	
Blood urea nitrogen (mg/dL)	16.09 ± 6.37	14.20 ± 4.71	14.96 ± 5.13	15.89 ± 5.69	19.12 ± 8.11	<0.0001
C-reactive protein (mg/L)	0.51 ± 0.92	0.41 ± 0.64	0.48 ± 0.96	0.50 ± 0.64	0.62 ± 1.25	<0.0001
Total protein (mg/dL)	7.24 ± 0.50	7.17 ± 0.49	7.25 ± 0.48	7.23 ± 0.48	7.32 ± 0.54	<0.0001
Total cholesterol (mg/dL)	211.47 ± 42.44	216.04 ± 40.28	214.59 ± 42.11	208.56 ± 42.49	207.19 ± 44.05	<0.0001
Serum phosphorus (mg/dL)	3.64 ± 0.54	3.68 ± 0.53	3.67 ± 0.56	3.60 ± 0.53	3.60 ± 0.55	<0.0001
Serum calcium (mg/dL)	9.51 ± 0.41	9.47 ± 0.40	9.53 ± 0.39	9.50 ± 0.42	9.53 ± 0.42	0.0078
Calcium supplementation (%)						<0.0001
Not use	55.90	48.87	52.46	58.43	63.10	
<0.4 g/d	23.29	22.11	22.88	24.01	24.05	
≥0.4 g/d	20.82	29.02	24.66	17.56	12.86	
Lumbar BMD (g/cm^2^)	1.01 ± 0.18	0.96 ± 0.17	0.99 ± 0.18	1.04 ± 0.18	1.06 ± 0.19	<0.0001

Mean ± SD for continuous variables: the *P* value was calculated by the weighted linear regression model. (%) for categorical variables: the *P* value was calculated by the weighted chi-square test. Abbreviation: BMD, bone mineral density.

**Table 2:** The association between serum uric acid (mg/dL) and lumbar bone mineral density (g/cm^2^).

	Model 1	Model 2	Model 3
	*β* (95% CI) *P* value	*β* (95% CI) *P* value	*β* (95% CI) *P* value
Serum uric acid (mg/dL)	0.030 (0.026, 0.034) <0.001	0.019 (0.015, 0.022) <0.001	0.010 (0.006, 0.014) <0.001
Serum uric acid categories			
Q1 (1.5–4.5 mg/dL)	Reference	Reference	Reference
Q2 (4.6–5.4 mg/dL)	0.034 (0.019, 0.050) <0.001	0.014 (−0.001, 0.029) 0.065	−0.004 (−0.018, 0.011) 0.627
Q3 (5.5–6.4 mg/dL)	0.081 (0.066, 0.097) <0.001	0.045 (0.030, 0.060) <0.001	0.017 (0.003, 0.032) 0.021
Q4 (6.4–13.7 mg/dL)	0.107 (0.091, 0.122) <0.001	0.060 (0.045, 0.076) <0.001	0.023 (0.007, 0.039) 0.004
Subgroup analysis stratified by sex			
Men	0.023 (0.017, 0.029) <0.001	0.021 (0.015, 0.027) <0.001	0.014 (0.008, 0.020) <0.001
Women	0.018 (0.014, 0.023) <0.001	0.019 (0.014, 0.023) <0.001	0.008 (0.003, 0.014) 0.001
Subgroup analysis stratified by race/ethnicity			
Non-Hispanic white	0.030 (0.025, 0.035) <0.001	0.020 (0.015, 0.025) <0.001	0.012 (0.006, 0.017) <0.001
Non-Hispanic black	0.020 (0.011, 0.029) <0.001	0.014 (0.005, 0.023) 0.002	0.007 (−0.003, 0.017) 0.164
Mexican American	0.025 (0.017, 0.032) <0.001	0.011 (0.004, 0.019) 0.004	0.010 (0.001, 0.018) 0.023
Other race/ethnicity	0.024 (0.010, 0.038) 0.001	0.015 (0.001, 0.029) 0.039	0.011 (−0.004, 0.025) 0.186

Model 1: no covariates were adjusted. Model 2: age, sex, and race/ethnicity were adjusted. Model 3: age, sex, race/ethnicity, waist circumference, education, income poverty ratio, physical activity, smoking behavior, alcohol consumption, blood urea nitrogen, C-reactive protein, total protein, total cholesterol, serum phosphorus, serum calcium, and calcium supplementation use were adjusted. In the subgroup analysis stratified by sex and race/ethnicity, the model is not adjusted for sex and race/ethnicity, respectively.

**Table 3:** Threshold effect analysis of serum uric acid on lumbar bone mineral density in non-Hispanic blacks using the two-piecewise linear regression model.

Lumbar bone mineral density	Adjusted *β* (95% CI), *P* value
Non-Hispanic black	
Fitting by the standard linear model	0.007 (−0.003, 0.017) 0.1643
Fitting by the two-piecewise linear model	
Inflection point	7.5
Serum uric acid <7.5 (mg/dL)	0.018 (0.004, 0.031) 0.0099
Serum uric acid >7.5 (mg/dL)	−0.026 (−0.055, 0.004) 0.0867
Log likelihood ratio	0.018

Age, sex, waist circumference, education, income poverty ratio, physical activity, smoking behavior, alcohol consumption, blood urea nitrogen, C-reactive protein, total protein, total cholesterol, serum phosphorus, serum calcium, and calcium supplementation use were adjusted.

## Data Availability

The survey data are publicly available on the Internet for data users and researchers throughout the world http://www.cdc.gov/nchs/nhanes/.

## References

[1] Wang P., Abdin E., Shafie S., Chong S. A., Vaingankar J. A., Subramaniam M. (2019). Estimation of prevalence of osteoporosis using OSTA and its correlation with sociodemographic factors, disability and comorbidities. *International Journal of Environmental Research and Public Health*.

[2] Ström O., Borgström F., Kanis J. A. (2011). Osteoporosis: burden, health care provision and opportunities in the EU. *Archives of Osteoporosis*.

[3] Perez-Ruiz F., Dalbeth N., Bardin T. (2015). A review of uric acid, crystal deposition disease, and gout. *Advances in Therapy*.

[4] Taghizadeh N., Vonk J. M., Boezen H. M. (2014). Serum uric acid levels and cancer mortality risk among males in a large general population-based cohort study. *Cancer Causes & Control*.

[5] Tana C., Ticinesi A., Prati B., Nouvenne A., Meschi T. (2018). Uric acid and cognitive function in older individuals. *Nutrients*.

[6] Huang X., Ng S. Y.-E., Chia N. S.-Y. (2018). Serum uric acid level and its association with motor subtypes and non-motor symptoms in early Parkinson’s disease: PALS study. *Parkinsonism & Related Disorders*.

[7] Du N., Xu D., Hou X. (2016). Inverse association between serum uric acid levels and alzheimer’s disease risk. *Molecular Neurobiology*.

[8] Wang L., Hu W., Wang J., Qian W., Xiao H. (2016). Low serum uric acid levels in patients with multiple sclerosis and neuromyelitis optica: an updated meta-analysis. *Multiple Sclerosis and Related Disorders*.

[9] Zhang F., Zhang Q., Ke Y. (2018). Serum uric acid levels in patients with amyotrophic lateral sclerosis: a meta-analysis. *Scientific Reports*.

[10] Kaushal N., Vohora D., Jalali R. K., Jha S. (2019). Review of the literature examining the association of serum uric acid with osteoporosis and mechanistic insights into its effect on bone metabolism. *Endocrine, Metabolic & Immune Disorders-Drug Targets*.

[11] Zhang D., Bobulescu I. A., Maalouf N. M. (2015). Relationship between serum uric Acid and bone mineral density in the general population and in rats with experimental hyperuricemia. *Journal of Bone and Mineral Research*.

[12] Dalbeth N., Topless R., Flynn T., Cadzow M., Bolland M. J., Merriman T. R. (2015). Mendelian randomization analysis to examine for a causal effect of urate on bone mineral density. *Journal of Bone and Mineral Research*.

[13] Lee Y. H., Song G. G. (2019). Uric acid level, gout and bone mineral density: a Mendelian randomization study. *European Journal of Clinical Investigation*.

[14] Curtin L. R., Mohadjer L. K., Dohrmann S. M. (2012). The national health and nutrition examination survey: sample design, 1999–2006. *Vital Health Stat*.

[15] Zhu Y., Pandya B. J., Choi H. K. (2011). Prevalence of gout and hyperuricemia in the US general population: the national health and nutrition examination survey 2007-2008. *Arthritis & Rheumatism*.

[16] Liu R., Han C., Wu D. (2015). Prevalence of hyperuricemia and gout in mainland China from 2000 to 2014: a systematic review and meta-analysis. *BioMed Research International*.

[17] Li X., Meng X., Timofeeva M. (2017). Serum uric acid levels and multiple health outcomes: umbrella review of evidence from observational studies, randomised controlled trials, and Mendelian randomisation studies. *BMJ*.

[18] Kubota M. (2019). Hyperuricemia in children and adolescents: present knowledge and future directions. *Journal of Nutrition and Metabolism*.

[19] Chen F., Wang Y., Guo Y. (2019). Specific higher levels of serum uric acid might have a protective effect on bone mineral density within a Chinese population over 60 years old: a cross-sectional study from northeast China. *Clinical Interventions in Aging*.

[20] Han W., Bai X., Wang N., Han L., Sun X., Chen X. (2017). Association between lumbar bone mineral density and serum uric acid in postmenopausal women: a cross-sectional study of healthy Chinese population. *Archives of Osteoporosi*.

[21] Dong X. W., Tian H. Y., He J., Wang C., Qiu R., Chen Y. M. (2016). Elevated serum uric acid is associated with greater bone mineral density and skeletal muscle mass in middle-aged and older adults. *PLoS One*.

[22] Chen L., Peng Y., Fang F., Chen J., Pan L., You L. (2015). Correlation of serum uric acid with bone mineral density and fragility fracture in patients with primary osteoporosis: a single-center retrospective study of 253 cases. *International Journal of Clinical and Experimental Medicine*.

[23] Babaei M., Shamsi R., Heidari B., Bijani A., Hosseini S. R. (2019). Serum uric acid status and its association with bone mineral density in the elderly people aged 60 Years and more. *International Journal of Endocrinology and Metabolism*.

[24] Ahn S. H., Lee S. H., Kim B.-J. (2013). Higher serum uric acid is associated with higher bone mass, lower bone turnover, and lower prevalence of vertebral fracture in healthy postmenopausal women. *Osteoporosis International*.

[25] Lee Y. J., Hong J. Y., Kim S. C., Joo J. K., Na Y. J., Lee K. S. (2015). The association between oxidative stress and bone mineral density according to menopausal status of Korean women. *Obstetrics & Gynecology Science*.

[26] Muka T., de Jonge E. A. L., de Jong J. C. K. (2016). The influence of serum uric acid on bone mineral density, hip geometry, and fracture risk: the rotterdam study. *The Journal of Clinical Endocrinology & Metabolism*.

[27] Pirro M., Mannarino M. R., Bianconi V. (2017). Uric acid and bone mineral density in postmenopausal osteoporotic women: the link lies within the fat. *Osteoporosis International*.

[28] Yan D.-d., Wang J., Hou X.-h. (2018). Association of serum uric acid levels with osteoporosis and bone turnover markers in a Chinese population. *Acta Pharmacologica Sinica*.

[29] Xiong A., Yao Q., He J., Fu W., Yu J., Zhang Z. (2016). No causal effect of serum urate on bone-related outcomes among a population of postmenopausal women and elderly men of Chinese Han ethnicity-a Mendelian randomization study. *Osteoporosis International*.

[30] Mehta T., Bůžková P., Sarnak M. J. (2015). Serum urate levels and the risk of hip fractures: data from the cardiovascular health study. *Metabolism*.

[31] Bonaccorsi G., Trentini A., Greco P. (2019). Changes in adipose tissue distribution and association between uric acid and bone health during menopause transition. *International Journal of Molecular Sciences*.

[32] von Elm E., Altman D. G., Egger M., Pocock S. J., Gøtzsche P. C., Vandenbroucke J. P. (2008). The Strengthening the reporting of observational Studies in epidemiology (STROBE) statement: guidelines for reporting observational studies. *Journal of Clinical Epidemiology*.

[33] Singh J. A. (2013). Racial and gender disparities among patients with gout. *Current Rheumatology Reports*.

